# Effects of Dynamic Suit Orthoses on the Spatio-Temporal Gait Parameters in Children with Cerebral Palsy: A Systematic Review

**DOI:** 10.3390/children8111016

**Published:** 2021-11-05

**Authors:** Natalia Belizón-Bravo, Rita Pilar Romero-Galisteo, Fatima Cano-Bravo, Gloria Gonzalez-Medina, Elena Pinero-Pinto, Carlos Luque-Moreno

**Affiliations:** 1 Department of Nursing and Physiotherapy, University of Cádiz, 11009 Cádiz, Spain; nataliabb1993@gmail.com (N.B.-B.); gloriagonzalez.medina@uca.es (G.G.-M.); carloslm@us.es (C.L.-M.); 2 Department of Physiotherapy, Biomedical Research Institute of Malaga (IBIMA), University of Málaga, 29071 Málaga, Spain; 3 Pediatric Rehabilitation Unit, Virgen del Rocío Hospital, 41013 Sevilla, Spain; fatima.cano.sspa@juntadeandalucia.es; 4 Department of Physiotherapy, Faculty of Nursing, Physiotherapy and Podiatry, University of Seville, 41004 Sevilla, Spain; epinero@us.es

**Keywords:** space suits, physical therapy modalities, cerebral palsy, gait disorders, neurologic, walking speed

## Abstract

Dynamic suit orthoses (DSO) are currently used as a complementary treatment method in children with Cerebral Palsy (cwCP). The aim of this review was to assess the effects of interventions with DSO on the altered spatio-temporal gait parameters (STGPs) in cwCP. An electronic search was conducted in the Web of Science, Scopus, PEDro, Cochrane Library, MEDLINE/PubMed, and CINAHL databases up to July 2021. We included a total of 12 studies, which showed great heterogeneity in terms of design type, sample size, and intervention performed (two employed a Therasuit, three employed the Adeli suit, three employed Theratogs, one employed elastomeric tissue dynamic orthosis, one employed a full-body suit, one employed external belt orthosis, and one employed dynamic orthosis composed of trousers and T-shirt). The Cochrane collaboration’s tool and the Checklist for Measuring Study Quality were used to assess the risk of bias and the methodological quality of the studies. It was variable according to the Checklist for Measuring Study Quality, and it oscillated between eight and 23. The studies of higher methodological quality showed significant post-intervention changes in walking speed (which is the most widely evaluated parameter), cadence, stride length, and step length symmetry. Although the evidence is limited, the intervention with DSO combined with a programme of training/physical therapy seems to have positive effects on the STGPs in cwCP, with the functional improvements that it entails. Despite the immediate effect after one session, a number of sessions between 18 and 60 is recommended to obtain optimum results. Future studies should measure all STGPs, and not only the main ones, such as gait speed, in order to draw more accurate conclusions on the functional improvement of gait after the use of this type of intervention.

## 1. Introduction

Cerebral palsy (CP) describes a group of permanent disorders of the development of movement and posture [1]. It is known as the most common physical disability in childhood [2,3,4]. The overall median prevalence estimate reported is 2.4 per 1000 live births [5]. Clinical diagnosis is based on a combination of clinical and neurological signs, such as spasticity, dyskinesia, epilepsy, musculoskeletal problems, behavioural disorders, intellectual disability, etc. [6,7]. Since the clinical presentation is very diverse, treatment is also a complex problem [8].

Children with CP (cwCP) show differences in mature gait development during the first eight months of independent walking (slower gait speed and greater individual support) [9]. Later, ambulatory cwCP demonstrate atypical gait patterns, and this impairment increases with increasing walking speed (fast walking speed) vs. preferred walking speed [10]. In addition, they have poor dynamic gait stability, as they require more strides to dissipate these gait disturbances, and, to compensate for these alterations, they show a greater stride width [10,11], modulating the length of their stride [12]. Despite the fact that this population presents greater variability and asymmetry in these spatio-temporal gait parameters (STGPs), some authors defend that this increase in speed does not result in a more unstable gait [13]. These asymmetries in STGPs correlate with a greater dependence on specific synergies in cwCP (fusion of synergies, excessive antagonist coactivation, and decreased muscle activation) [14]. Among these parameters, the maximum step length of the dominant side appears to be crucial for walking efficiency in cwCP, which can be very useful in reducing fatigue and allowing these children to walk for longer periods [15]. A decrease in speed, stride length, step length, and monopodal stance time has been observed, as well as an increase in cadence, step width, and double support time in the gait of cwCP [16,17]. Likewise, different studies have evaluated the relationship of these parameters with the level of functionality, revealing the existence of significant correlations with the performance of balance (anticipatory and postural responses) and the risk of falls [18,19]. The decline of movement coordination and the presence of posture disorders in cwCP lead, over time, to the appearance of orthopaedic problems that negatively influence the health and functional state of the individual, limiting and disabling activities such as gait [20].

One of the most important functional goals of physical therapy is to increase walking speed. To date, functional gait training [21] appears to be the most effective treatment modality, whereas strength training has been proved to be less effective [22], showing additional benefits with speed training, biofeedback training, whole body vibration [23,24], virtual reality [21], electrical stimulation, and transcranial stimulation [24]. Exercise interventions also appear to have a beneficial effect on gait speed, although without significant effects on gross motor function in cwPC, which does seem to improve with aerobic exercise (although with no improvement in gait) [22] and robotic gait training [25,26]. Treadmill training in cwCP is also effective for gait speed, gait endurance [27], and limb support time, with no significant effects on cadence or stride length [28]. BoNT-A and/or casting, orthopaedic devices, strength training, balance/gait training, and individualised therapy also significantly changed [29] STGPs in children with bilateral spastic CP. Therefore, throughout history, different types of orthotic devices have been used to prevent the appearance of deformities, correcting or reducing those that are already present, and ease gait function by stabilising the lower extremity (LE) in three dimensions of space [30].

The most recent model of dynamic orthosis is known as Dynamic Suit Orthosis (DSO), and its use as a treatment protocol is called “suit therapy”. In the literature, two possible forms of intervention with the DSO are described: single use of the suit [31,32,33] or use of the suit within a personalised intensive training programme composed of functional exercises and muscle-strengthening exercises performed in units of skill exercise [34,35]. Therefore, as can be seen, there is no single standardised protocol in suit therapy, which has led to the emergence of different methods, the best of which are, currently, the Therasuit, Adelisuit, Pediasuit, and Upsuit methods [36]. Each method establishes a different intensive therapy protocol, consisting of long-lasting sessions, distributed over a number of sessions per week, over a period of several weeks. Some of them also use body support elements such as elastic cords and a metal cage (SPIDER system) to help verticalisation when the child is unable to stand upright on his or her own [37].

Despite the importance of specifically assessing and treating gait disturbances, it is worth remembering the paradigm shift that has taken place in the therapeutic approach to treat cwCP and other disabilities since the publication of the International Classification of Functioning, Disability, and Health (ICF) [38]. Especially in the paediatric age group, it should be emphasised that the treatment of a given condition should encompass aspects that are not only related to a specific activity, such as gait. In this way, the WHO created the ICF-CY for children and young people [39]. In this regard, the best available intervention evidence for cwCP is described in a systematic review published by Novak et al. in 2013 [40] and updated in 2019 [41]. A high percentage of interventions commonly used for the treatment of cwCP have not generated sufficient evidence to support their use. Such is the case of soft dynamic orthotic full-body suit [40]. However, papers published before this systematic review of 2013, 2019, and those published thereafter show contradicting opinions about this kind of intervention [41,42,43]. Therefore, it is interesting to study in depth specific functions, structures, and parameters, such as those involved in gait, in order to be able to relate the different domains of ICF in further studies. Only in this way will there be data with which to work objectively, bearing in mind that the ultimate goal must always be to improve the independence of these children. Furthermore, it is also interesting to know in detail the results obtained in studies with different devices to which families have access in order to report on specific aspects that have not been addressed in previous reviews.

On the other hand, neuroplasticity is harnessed through high-intensity or high-dose training. Given the costs and time burden for families of cwCP, it is important to quantify which rehabilitation training approaches and doses confer the largest clinical gain [44], and some reviews are published about this topic [36,45,46,47]. Although suit therapy does not appear to significantly improve functionality in cwCP, its use as an adjunctive treatment and always supervised by a professional provides promising results in improving proximal stability [47]. Therefore, this study provides new evidence on the use of suit therapy in the improvement of gait parameters in cwCP.

The interest in this review arose after carrying out an exhaustive search of the databases on the evidence of the effects of the intervention with DSO in cwCP and finding that the outcome “gait”, in a global way, has been one of the most studied and committed in this respect. Thus, recent reviews show controversial results, and their authors suggest the need for new research to clarify their usefulness and effectiveness in treatment. However, despite the lack of solid evidence and its use, which involves a significant expenditure of money and time, DSOs are currently being used in rehabilitation services with encouraging results. For these reasons, we believe that it is extremely important to evaluate the effects and utility of the use of DSO in the management of gait disturbances, especially in its STGPs, since they are closely related to energy cost, functional impairment, and efficiency in ambulatory capacity [48]. This review aims to contribute to the decision of the scientific community to estimate or disapprove its inclusion in the treatment protocol for this disorder. The aim of this study was to analyse the effects of DSO on the STGPs in cwCP and to clarify its efficacy and usefulness in the treatment of these disorders.

## 2. Materials and Methods

### 2.1. Study Design

The present study carried out a systematic review of the literature according to the Preferred Reporting for Systematic Reviews and Meta-Analyses (PRISMA) [49] standards and started the protocol for registration in PROSPERO (CRD42019139339).

### 2.2. Search Strategy

An electronic search of articles currently published in the Web of Science, Scopus, PEDro, Cochrane Library, MEDLINE/PubMed and CINAHL databases was performed up to July 2021 by two independent reviewers (NBB and CLM); a third reviewer (RPRG) resolved all the discrepancies that emerged between the two independent reviewers. The equations for Search terms employed arose from the combination of uncontrolled terms (such as suit wear, Lycra, Adeli, Therasuit, strapping system, Theratog*, suit therapy) and MeSH terms (such as Space Suits, Cerebral Palsy, and Gait) (Table 1). The terms dynamic elastomeric, orthotic garment, therapeutic vest, and external strap orthoses were not included in the search strategy, as they were not relevant to the results. We also searched other sources manually in Google Scholar and in the reference lists of the articles found to identify and include potentially relevant studies in this review. No filters were applied in terms of time or language limitation.

### 2.3. Inclusion and Exclusion Criteria

The topic chosen for this review is quite novel; therefore, a heterogeneous group of experimental designs was included, such as randomised clinical trials (RCTs), in order to obtain further evidence, given the scarcity of RCTs. In addition to these types of studies, we included quasi-experimental designs, case series, and single-subject experimental designs, following the PICO model [50] (Population, Intervention, Control/Comparison and Outcomes). All studies that met the following criteria were included in the review:

- P (Patients): cwCP with Gross Motor Function Classification System (GMFCS) level I–III.
- I (Intervention): Use of full body DSO. Interventions were included if they consisted in wearing the suit in isolation or using the suit in combination with conventional physical therapy (Neuro-Development Treatment, exercise programme). Theratogs, Dynamic Elastomeric Fabric Orthosis (DEFO) trousers, and external strap orthoses were included for their impact on pelvic biomechanics and axial control.
- C (Comparison): no intervention, other interventions, no comparison, and additional effect of DSO with conventional physical therapy.
- O (Outcomes): Spatio-temporal gait parameters.

Similarly, those articles that met some of the following criteria were excluded:

- Use of subjective tools for measuring results (observational analysis, etc.).
- DSO intervention combined with other non-conventional therapies (i.e., virtual reality or use of robotic devices), except for those performed with the use of the suit.

### 2.4. Assessment of the Methodological Quality, Level of Evidence, and Grade of Recommendation

The methodological quality of the studies was evaluated using the Checklist for Measuring Study Quality (CMSQ), which has shown to be a valid and reliable instrument for evaluating randomised and non-randomised studies, obtaining the respective correlation values for criterion validity and intra- and inter-examiner reliability (r = 0.90, 0.88, and 0.75) [51]. The CMSQ is currently recommended in systematic reviews that include different types of experimental designs [52]. This checklist consists of 27 questions grouped into four main dimensions: study quality (10 items), external validity (3 items), internal validity (13 items), and statistical power (1 item). All items have a score of 0 or 1, with the exception of item 5, which allows scores of 0.1 or 2, and item 27, which establishes scores from 1 to 5, resulting in a maximum score of 32 points. From this, Downs and Black indicated that the presence of subscales would allow researchers to interpret the particular methodological strengths and limitations of each study [51].

The level of evidence and grade of recommendation was evaluated through the classification proposal of the Centre for Evidence-Based Medicine of Oxford (OCEBM) [53], which is highlighted as one of the most widely used evidence ranking systems in the field of health due to its high degree of specialisation according to the type of clinical setting [54]. In this way, the OCEBM allows evaluating the evidence according to the subject area and type of design, granting different degrees of level of evidence to each type of study according to the relevant clinical setting and establishing different degrees of recommendation (A, B, C, and D) to each of them according to the level to which they belong [55].

The assessment of the risk of bias of RCTs was performed through The Cochrane Collaboration´s tool, using the Review Manager (RevMan) (Version 5.4, Cochrane Training, London, UK) [56]. This tool allows the evaluation and the rating of the risk of bias in terms of “low risk”, “high risk”, and “unclear risk”.

## 3. Results

**Figure 1:**
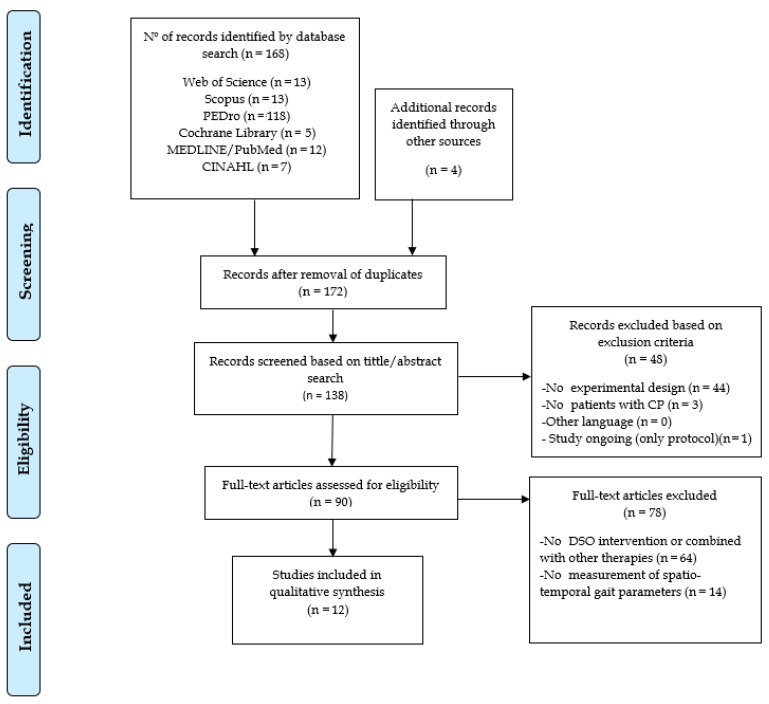
Flow diagram of the article selection process according to the PRISMA standards. (CP) Cerebral palsy; (DSO) Dynamic suit orthosis.

From a total of 172 studies initially obtained, only 12 studies were selected for the elaboration of the systematic review (four RCTs, three quasi-experimental designs, three case series, and two single-subject experimental designs). Figure 1 shows the complete selection process.

Table 2 shows the summary of articles included in the systematic review.

### 3.1. Methodological Quality, Level of Evidence, Grade of Recommendation, and Risk of Bias

**Figure 2:**
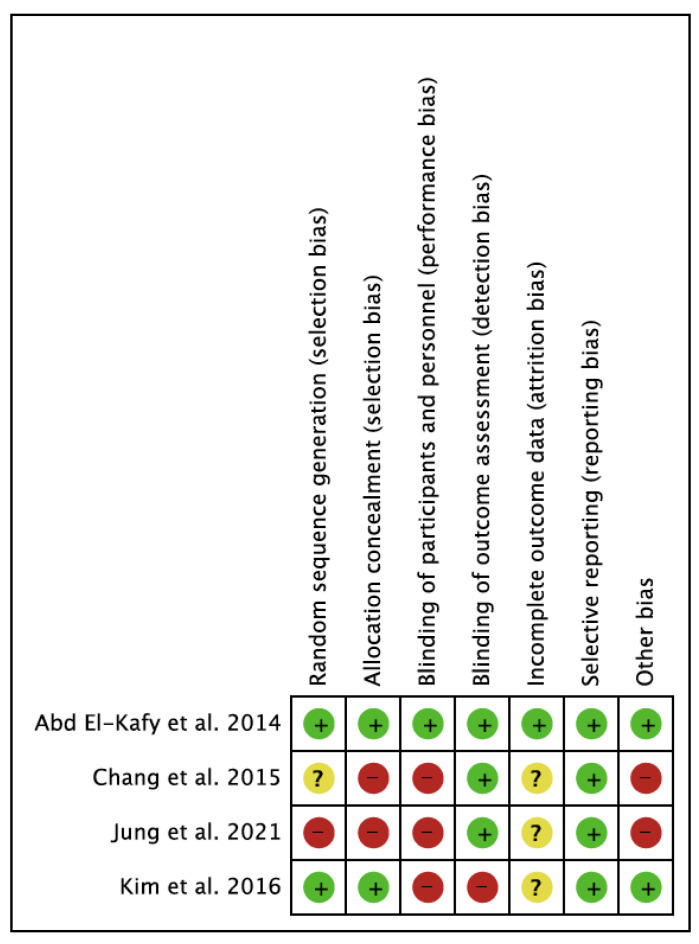
Risk of bias of the studies included in the systematic review. In green: low risk of bias; yellow: unclear risk of bias; in red: high risk of bias.

**Figure 3:**
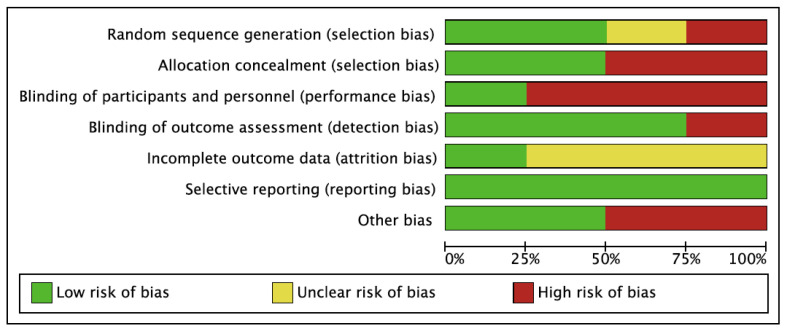
Overall risk of bias. The results are presented by percentages.

The methodological quality reported by the Checklist for Measuring Study Quality for the 12 studies was highly variable, with a mean score of 15.2 and a total range of 8 to 23 (Table 3). In general, the best scores were obtained in RCTs (23, 22, 21, and 19). Regarding the risk of bias of the studies, the study conducted by Abd El-Kafy et al. [62] presented the lowest risk of bias. In terms of the risk of bias among the studies, the lowest bias was obtained in the selective reporting (reporting bias). The highest value was found in the blinding of participants and personnel (performance bias). The results are shown in Figure 2 and Figure 3.

### 3.2. Participant Characteristics

A total of 158 subjects (aged 3–14 years) were studied. All samples included cwCP, except one study that used typically developing children in the comparison group [33]. Nevertheless, only cwCP were considered for the review. GMFCS was used to quantify the degree of motor disability and was specified in all studies, with values of I–III. It is worth noting the large variability in sample size, ranging from one to 57 participants; however, the distribution and clinical phenotype of CP was quite homogeneous, with spastic diplegia being the most frequent type, except in Kim et al. [59] and Christy et al. [35], who used more diverse phenotypes.

### 3.3. Intervention Characteristics

Regarding the dynamic orthosis models used, the methodological quality of the studies that evaluated the effects of wearing the Adeli suit obtained a percentage of 71%, 34%, and 25% [59,60,63], 59%, and 40% in studies with a Therasuit [34,35], 68%, 59%, and 37% with Theratogs [31,57,62], 65% with external strap orthosis [61], 43% with DEFO [32], 34% with a full suit [63], and 28% in dynamic shirt and pants orthosis [58]. After evaluation with the Oxford scale, seven studies were obtained with C, 4; four studies were obtained with A, 1b; and one study was obtained with B, 3b. The interpretation of these studies revealed a high level of evidence and a strong recommendation for the use of the Adeli suit, Theratogs, and external strap orthosis for the treatment of the following spatio-temporal variables: walking speed, cadence, stride length, step length, and single-double support time. The use of a full-body suit obtained a moderate level of evidence and favourable recommendation for the treatment of walking speed and cadence [33]. Finally, DEFO, Therasuit, and dynamic T-shirt and pants presented poor evidence and a favourable but inconclusive recommendation for use in managing walking speed, cadence, single-double support time, and step length [32,34,35,58].

The interventions used in the included articles were very varied. Thus, some interventions consisted in simply wearing the dynamic suit orthosis for a certain time, others combined the use of the suit with intensive exercise programs or neurodevelopmental treatment, and other studies only aimed to evaluate the immediate effects on gait parameters that occurred upon donning the suit [59]. Programme doses ranged from 30 min to 12 h/day [31,59], with a frequency of one to five sessions/week [31,32,63] and a total duration of the programme from three to 18 weeks [34,35,63]. The time of isolated use of the suit as a single intervention ranged between 10 and 30 min [33,57,61]. A wide range of types of dynamic orthoses were used; thus, two studies used a Therasuit [34,35], another three used the Adeli suit [59,60,63], three used Theratogs [31,57,62], one study used the DEFO pants [32], one used the full-body suit [33], one used dynamic orthosis composed of a T-shirt [58] and pants, and another study evaluated the effects of the external strap orthosis [61]. The intervention protocols varied between the studies and among the participants themselves to set up individualised therapeutic programmes attending to specific needs; thus, balance, walking, climbing stairs, sitting and standing, jumping, resistance, motor control, and coordination exercises were used. In other cases, the suit was used as part of the Therasuit method [34,35], which was preceded by a preparation period that included a few minutes of warm-up, massage, and stretching. Only one study evaluated the additional effect of the suit therapy on the neurodevelopmental treatment [59]. Likewise, the external strap fastening system varied in each individual according to the functional limitations that he/she presented, being mostly used in cases of spastic diplegia to correct squatting by activating the external rotator, hip abductor, knee extensor, abdominal, oblique, trunk extensor, and interscapular muscles.

Most studies that employed exercise programmes performed the post-intervention measurement without the suit, except Skublewska-Paszkowska et al. [58], who measured the effects after the intervention with and without the suit. On the other hand, mostly, the immediate effects after the intervention were evaluated, and only a few studies followed up the residual effects of the intervention in the medium–long term [31,32,34,35].

### 3.4. Outcomes Measures

The reported results were analysed according to the types of intervention performed (simple use of dynamic orthosis or combined with an exercise programme), dose of applied intervention (single, medium-duration programme: 3–6 weeks; long-term programme: 12–18 weeks) and timing of outcome measurement (immediate: after withdrawal of intervention; short-term: 4–6 days later; long-term: 6–18 weeks later). The effects observed on each of the variables with the different types of dynamic suit orthoses and types of intervention are detailed below.

Gait speed was the variable analysed par excellence, being evaluated in 11 of the 12 selected studies. Most of the studies used three-dimensional analysis of movement for their evaluation, except for two studies that used the 10MWT [32,63]. A significant improvement of this parameter was observed after the intervention with the seven DSO models. However, the results differed according to the type of intervention. Thus, two authors reported a significant immediate increase when measuring while wearing the full-body suit (0.07 m/s) [33] or during the use of a spiral-applied external strap orthosis on the LEs (0.03 m/s) [61]. Three studies evaluated the changes in this parameter after interventions consisting in the isolated use of the suit, the use of DEFO for 6 weeks [32], and Theratogs for 12 weeks [31], showing no significant changes, although Jung et al. did report improvements after 30 min of full-body Theratogs (0.26 m/s). With respect to suit interventions combined with exercise, two studies using medium-duration programmes reported significant effects with Adeli suit use (0.14 m/s and 0.05 m/s) [59,60], and only Bailes et al. [34] reported sustained improvement in the short term (4 days later) after application of a 3-week programme with Therasuit (0.17 m/s). Three studies evaluated the immediate effects after a long period of intervention. Ko et al. [63] obtained an improvement of 0.29 m/s with the use of the Adeli suit in an 18-week functional exercise programme and an improvement of 0.05 m/s after using the Theratogs in a standing and gait exercise programme. However, the best results for this variable were obtained after an exercise programme performed with the shirt and pants orthosis for 3 months and the immediate measurement wearing the suit (0.31 m/s) [58,62]. Only Matthews et al. [32] and Flanagan et al. [31] evaluated the permanence of the long-term effects, reporting a decrease in velocity 6 weeks after the DEFO pants intervention phase, with a remaining small effect of reduced parameter variability with respect to the pre-intervention measurement. Nevertheless, the Theratogs model showed no permanence of effects 2 and 4 months after the intervention [31].

Cadence was the second most examined variable; it was analysed in most of the studies that evaluated walking speed due to the strong relationship between these two parameters. Those studies used three-dimensional analysis of movement for evaluation, except for one that used a step activity monitor [35]. The effects on this variable were evaluated by six of the seven DSO models, since no study evaluated the changes with the DEFO pants model [32]. Of these six DSO models, all registered significant changes in cadence, with the exception of the shirt-and-pants orthosis model [58]. The measurement wearing external strap orthoses and a full-body suit showed immediate improvements in cadence (2.45 step/min and 12.1 step/min, respectively) [33,61]. Interventions consisting in the isolated use of Theratogs (30 min) [57] reported the greatest increase in the number of steps (27.94 steps/min), except for those in which the suit was used for a long period [31]. Medium-duration exercise programmes using Adeli combined with the Bobath concept for 6 weeks obtained an increase of 12.96 step/min [59], and its use with functional exercises for 4 weeks also showed good results, although less striking (3.05 step/min) [60]. However, the Therasuit programme improved the number of steps in the short term in one of two studies that used this model [34]. Abd El-Kafy performed the only long-duration (12 weeks) exercise programme, which showed improvements in this parameter with both the isolated use of Theratogs (5.83 step/min) and the combined use with ankle foot orthoses (10.92 step/min) [62]. No studies reported long-term effects.

Stride length was examined by eight of the 12 studies through the three-dimensional movement analysis system, although only three studies reported significant results [61,62,63]. Immediate measurement after a long-term intervention programme of 18 weeks employing the Adeli suit obtained the most significant change (21 cm) [63], followed by a 12-week programme using Theratogs, which showed an increase of 15 cm [62]. However, medium-duration programmes employing both the Therasuit and Adeli models reported no significant changes [34,59,60]. Interventions of isolated use of the suit were only performed with the Theratogs model, with no significant findings [57]. Nevertheless, the measurement of this parameter increased immediately when wearing the external strap orthoses (3 cm) [61] but not with the full-body suit model [33]. Regarding the prevalence of the effects achieved, all results were obtained immediately, and no study showed permanence of these changes in the short or long term.

The step length parameter was evaluated in seven of the 12 studies with five different DSO models using 3D motion analysis, obtaining the least positive results in relation to the number of models that evaluated it. Studies using a full-body suit and external strap orthoses did not report immediate changes with suit fitting [33,61]. On the other hand, isolated suit use interventions only reported post-treatment changes with the use of full-body Theratogs in a 30-min session (4.92 cm and 5.34 cm in each LE) [57]; nevertheless, prolonged use for 82 h/12 weeks showed no appreciable immediate change or permanence of any long-term effect [31]. The use of the Adeli suit combined with Bobath or functional exercises in medium-duration programmes (4 and 6 weeks, respectively) reported no increase in step length, although it did show improvements in step symmetry [59]. However, a study in similar conditions with the use of a Therasuit showed no improvement [34]. The study of Skublewska-Paszkowska et al. [58] was the only one to employ a long-term programme (3 months) combining T-shirt and pant orthoses with static-dynamic exercises, reporting stabilisation of the parameter at post-intervention measurement with the suit (an increase of 7.8 cm). The data analysis revealed positive effects only for this variable after immediate post-intervention measurement, as studies analysing short- and long-term effects did not report significant changes [31].

The variables walking time, stance time, and stride width were analysed by a minority of studies. Step time was evaluated wearing shirt-and-pants orthoses, showing no significant changes [58]. However, stride time was assessed after an 18-week Adeli suit programme, showing a significant reduction of 3.64 s [63]. Within the stance time in the gait cycle, the single support time and double support time was analysed. Thus, Jung et al. [57] showed immediate changes with the use of the full-body Theratogs in both parameters (0.41, 0.33 in single support and 0.18 in double support). In contrast, Bailes et al. [34], with a 3-week Therasuit programme, only recorded isolated changes in single and double support time in a single subject. Finally, stride width was only analysed by Ko et al. [63], who recorded a 1 cm change in a single LE when evaluated after a long-duration (18 weeks) intervention programme using the Adeli suit combined with relaxation, strengthening, and gait exercises.

One study reported improvements in walking speed with the use of DEFO [32], another study observed improvement in speed and cadence as well as decreased time in double support after the intervention with Therasuit [34], one article noted improvements in speed, cadence, and stride length after the use of Theratogs [62], and another article reported benefits only in speed and cadence with the use of the full-body suit [33]. Three studies found benefits in the speed, cadence, and stride length with the use of the Adeli suit [59,60,63], two of which also reported an improvement in step length asymmetry [59,60]. One study reported improvements in stride length, speed, and cadence after the use of an external strap orthoses [61], and another study reported improvements in speed and step length with the use of a dynamic shirt-and-pants orthosis [58].

Speed, cadence, and stride length were the spatio-temporal parameters that registered the greatest significant changes in the evaluated studies. A single study recorded significant results in step length, reduction in double support time, and step width [63]. It was also observed that the temporal variable rolling time was not evaluated by any of the included studies.

## 4. Discussion

This systematic review evaluated the effects of using DSO (isolated or associated with a training programme) on the STGPs in cwCP. In the selected studies, seven different DSO models were used, showing a great variability of results for each of the analysed variables, thus making it necessary to set up a comparative analysis of the study variables and the interventions carried out to answer the research questions raised.

### 4.1. Outcome Measures, Intensity, and Type of Intervention

All the investigations examined the changes in the spontaneous gait speed, which could be due to the fact that it is the most functional parameter in these individuals, as it has a lower energy cost [64]. This parameter experienced significant changes after the interventions of isolated use of the suit for short [57] and long periods (12–18 weeks) [63], and it presented worse results in the medium-duration interventions (3–6 weeks) [59,60]. Based on the total number of sessions used in the programmes, the most significant changes were detected in gait speed after 18 sessions [63].

For cadence, the greatest significant changes are in the interventions of isolated use of the suit (short duration) [33,57], and it also showed a significant increase in immediate measurement in medium-duration programmes, highlighting an exponential increase in this parameter for a programme with a large number of sessions (n = 60) [59]. However, the changes detected in this variable were not always interpreted as positive effects on the individual, since, in some cases, this increase was associated with a gait strategy consisting in a greater frequency of steps (cadence), to counteract a shorter step and stride length in order to achieve the desired speed [65], although at the expense of a higher energy cost [66].

Based on the referenced data analysis, stride length showed mainly significant changes associated with long-duration interventions (12–18 weeks) [62,63]; only one study showed immediate improvements with external strap orthoses [61], although the changes in the variable were small, and no improvement was obtained in medium-duration interventions. Stride length also showed the best results after 18–60 sessions as well as walking speed [63].

The results only reflected significant improvements for an isolated intervention with a suit [57]; however, a normalisation of the asymmetry of the step, together with an increase in the walking speed and cadence, was also contemplated after interventions of medium-long duration (4, 6, and 12 weeks) [58,59,60]; thus, the joint interpretation of the variables seemed to lead to positive functional changes for the individual. Few studies evaluated the effects of combined DSO exercise programmes on step length and reported only significant results for decreased step asymmetry after a variable range of 20–60 sessions [59,60].

The variables walk time, support time, and stride width were examined in a minority of studies; thus, it was not possible to compare the results for different intervention doses, despite having shown a strong relationship with dynamic balance during ambulation and their implications on gait performance [18]. Double support time and stride width after 15–18 sessions showed the best changes [34,63], although these two variables were only examined by a minority of studies, and the results could not be contrasted.

The analysis of the data recorded for the different STGPs outcomes after the intervention with the different DSO models revealed a clear effect on the speed, cadence and, to a lesser extent, stride length, and it reported certain modifications in the asymmetry of step length and time in single and double support and stride width; however, the number of studies that reported positive results was smaller than for the other parameters, and therefore, it would be necessary to continue investigating the effects on these outcomes. Thus, for the interpretation of the results, the joint evaluation of the analysed spatio-temporal outcomes was necessary. Most of the analysed variables reported immediate positive post-intervention effects, in such a way that only the shortest double support time, speed increase and cadence prevailed days after withdrawal from the intervention, and no long-term effects were observed for any of the parameters [34].

On the other hand, in long-duration programmes, it was observed that the increase in speed and cadence followed a similar path, which could be related to a true functional improvement in the individual [62] without losing stability in cwCP with high levels of functionality (I-II GMFCS) [13].

The presented analysis revealed a greater effect on changes in walking speed, cadence, stride length, and step length asymmetry in cwCP after medium-long programme interventions and suit removal [59,63], with some exceptions [58]. Likewise, the same parameters manifested an immediate significant effect with a single dose and single use of the suit; however, the intervention used was of short duration (30 min), and the permanence of the effects is unknown [57]. Furthermore, in some studies, the results were obtained while the individual was wearing the DSO model, and it is unknown whether the effects prevailed after removal of the suit [33,61].

The DSO intervention combined with an exercise/physical therapy programme showed the greatest significant changes for most of the parameters studied, with the exception of the study by Jung et al. [57], who only used the suit, showing similar results. Likewise, the step time variable was evaluated by a single study without significant results [58]; support time and stride amplitude did show changes in only one study that evaluated them [34,63]. Similarly, different effects were observed on the parameters evaluated according to the type of exercises included in each programme with DSO, with the best results being obtained in those that used dynamic balance, coordination, walking, sitting, standing, and stairs exercises [34,59,63]. Likewise, the programmes that achieved greater positive modifications in the STGPs alterations used the Therasuit and Adeli suit, according to established intervention protocols, which shared similarities in the planning of the sessions (1st stage: preparation and relaxation; 2nd stage: adaptation to the suit; and 3rd stage: carrying out exercises), standard application of straps connecting vest–pants, pants–knees, and knees–shoes, application of techniques to achieve muscle tone inhibition and postural correction, use of a short period of time (5–10 min) for assimilation and adaptation to the suit, and carrying out exercises focused on functional objectives (15–30 min). Furthermore, the studies that obtained the best results reported the participation of trained physiotherapists in the programme and highlighted the importance of their experience and degree of training [34,63], since, in some cases, they had to intervene in the postural correction during gait using manual assistance [63], and were in charge of adjusting the different tensions to the external straps and including some additional straps according to the deficits and functional needs observed in each individual [34].

The heterogeneity of doses and intervention protocols used with the different DSO models, as well as the absence of studies investigating the effects of some models on certain parameters made the analysis difficult in this review; however, we observed a number of trends for further research in future clinical trials.

### 4.2. Functional Implications

CP gait is normally characterised by presenting a compensatory strategy consisting of increasing the number of steps/minute to counteract a shorter step length and stride, which could be associated with alterations in muscle tone [67] and thus be able to achieve speed [18], although at the cost of higher energy consumption [66]. The results obtained after the analysis of the selected studies suggest possible positive effects after the intervention with DSO on the alterations found in the average speed, cadence, stride length, and step length asymmetry during walking in cwCP. In turn, these modifications could lead to a series of functional benefits, since several studies corroborate the relationship that these parameters have with the level of physical function, gait performance, and quality of life in these individuals [18,68]. Thus, spontaneous gait speed has shown a significant and positive correlation with the level of anticipatory response and postural adjustment reactions measured in these individuals through the test of balance measurement systems [BESTest (developed by Dr. Horak)] [18] and with the functional level of the LEs evaluated through the short physical performance battery (SPPB) [68] as well as a negative correlation with the risk of falling during walking [18]. Numerous studies have reported the relationship of cadence and step length with gait performance, observing that the latter has a stronger relationship with energy expenditure and fatigue [15]. Although only one intervention with DSO from the included studies revealed effects on step length, statistically significant differences were found for variability in step length, whose improvement has also been related to a lower risk of falling [18]. Likewise, the effects on step length after the use of exercise programmes with DSO were evaluated by a minority of studies, which may have not used the most appropriate exercises to obtain gains in this parameter, since, in other investigations, there is evidence of improvement in the magnitude of this variable after specific hip and knee exercise programmes [15]. The outcomes support time and “stride width” have also been shown to be important in the management of changes in dynamic balance and risk of falling [18,67], although only one study with DSO reports improvements in these parameters; therefore, further research is needed to clarify the effects on these variables. Therefore, based on this analysis, it could be suggested that these STGP variables are part of an important therapeutic target in the clinical management of the functional alterations observed in these individuals.

### 4.3. Methodological Quality

In general, all the studies scored well in the study quality dimension, except for the confounding factors, adverse events, and loss to follow-up sections, which were reported only by a minority of them. Internal validity was moderate for most studies, since only four had randomised groups, and, considering the nature of the treatment, blinding of the participants and evaluators was very difficult, in addition to the fact that few studies provided information regarding the joint origin of the sample, the period of participant recruitment, and the adjustments of follow-up lengths used. External validity was poor in all studies, as none proved that the patients in the sample were representative of the population by not reporting on the distribution of confounding factors.

Regarding the dynamic orthosis models used, the methodological quality of the studies that evaluated the effects of wearing the Adeli suit obtained a percentage of 71% [59], 34% [63], 25% [60], 59% [35], and 40% [34] in studies with Therasuit, 68% [57], 59% [62], and 37% [31] with Theratogs, 65% with external strap orthosis [61], 43% with DEFO [32], 34% [63] with full suit, and 28% [58] with dynamic shirt-and-pants orthosis. After evaluation with the Oxford scale, seven studies were obtained with C, 4; four studies with A, 1b; and one study with B, 3b, whose interpretation revealed a high level of evidence and a strong recommendation for the use of the Adeli suit, Theratogs, and external strap orthosis for the treatment of the spatio-temporal variables walking speed, cadence, stride length, step length, and single-double support time. The use of the full-body suit obtained a moderate level of evidence and favourable recommendation for the treatment of walking speed and cadence [33]. Finally, the DEFO suit [32], Therasuit [34,35], and dynamic T-shirt and pants [58] presented poor evidence and a favourable but inconclusive recommendation for use in managing walking speed, cadence, single–double support time, and step length.

The studies of higher methodological quality suggested that cwCP with level I–II GMFCS could improve the alterations in speed, cadence, and stride length after medium- and long-duration interventions with the Adeli and Theratogs models combined with an exercise programme [59,62] and immediately by simply using the external strap orthosis [61]. Similarly, improvements in step length asymmetry were also reported after the 6-week intervention with the Adeli model [59]. According to the Oxford scale [54], the DEFO suit, T-shirt-and-pants, and Therasuit models obtained poor evidence and a favourable but inconclusive level of recommendation for the treatment of changes in speed, cadence, step length, and time in double support.

These findings suggest a cautious interpretation of the results of the studies included in this review. The same problem is mentioned in other reviews carried out on the use of these devices in cwCP [36,45,46,47]. The benefits from the treatment should be assessed via ICF [38,39]. The success of an intervention can be defined as its capacity to promote functioning and ease disability [36]. Our research provides results focused on gait parameters. This implies not only the approach of the structure and the function but of the activity following the paradigm of the ICF [38].

### 4.4. Limits and Future Research

Some limitations can be mentioned, such as the restrictions of language (only studies published in English and Spanish) and databases (Web of Science, Scopus, PEDro, Cochrane Library, MEDLINE, and CINAHL). The main difficulties were observed in the heterogeneity of the included study designs due to the scarce number of articles found and the absence of research with high methodological quality. From the present review derives the need to continue developing studies of high methodological quality on this topic that evaluate the effects of the DSO on the STGP already evaluated, in addition to the swing time, support time, and stride width, since these are especially related to the risk of falling in the individual, and the positive results could condition important functional changes. Finally, future investigations are encouraged to compare the effects achieved with different DSO models on the STGP disturbances in cwCP after being applied with the same exercise programme and intervention dose. There is no standardised protocol for the use of this type of device, and these limitations should be considered when advising parents [41,46]. Therefore, although suit therapy is not recommended as a stand-alone treatment, it would be very important to recognise it as an add-on treatment for some families. The process of donning a suit may mean they engage in more intensive therapies and active practice, which may produce positive results [41]. There is also a need for further research including long-term follow-up of participants to see if the results obtained are sustained over the years as in other studies on cwCP gait [69].

## 5. Conclusions

Although the evidence is limited, DSO has positive effects on the STGP alterations in cwCP, especially in speed, cadence, and stride length. Despite the good results obtained by some authors with measurements of the STGP during the intervention or after a single session, better results were shown after interventions combined with programmes of medium-long duration (18–60 sessions). The changes described suggest functional repercussions in cwCP (better dynamic balance, greater LEs functionality, increased efficiency during walking, and decreased risk of falling), thus improving the quality of life of the individual. All the dynamic orthosis models reported some beneficial effect on the STGP, improving even more when combined with training/physical therapy programmes. The results of Therasuit and the Adeli suit stand out, with well-defined intervention protocols. The use of DSO in cwCP has been proved to be safe, with no adverse effects, and it could be an optimal complement to physiotherapy. However, future RCTs that explicitly include these variables are necessary, evaluating all the STGPs of gait and not only the main ones, such as gait speed, since this would help to discern whether the improvement in gait speed does not entail associated compensatory mechanisms that lead to a less functional gait.

## Figures and Tables

**Table 1:** Search strategy used in the different databases.

Databases	Search Equation	Results
Web of Science	TS = (“suit wear” OR “space suits” OR lycra OR Adeli OR therasuit OR “strapping system” OR theratog* OR “suit therapy”) AND TS = (“cerebral palsy” AND gait)	13
Scopus	TITLE-ABS-KEY ((“suit wear” OR “space suits” OR lycra OR Adeli OR therasuit OR “strapping system” OR theratog* OR “suit therapy”) AND (“cerebral palsy” AND gait))	13
PEDro	Therapy: orthoses, taping, splinting Subdiscipline: neurology Topic: cerebral palsy	118
Cochrane Library	TITLE-ABS-KEY (“suit wear” OR “space suits” OR lycra OR Adeli OR therasuit OR “strapping system” OR theratog* OR “suit therapy”) AND “cerebral palsy” AND gait	5
CINAHL	(“suit wear” OR “space suits” OR lycra OR Adeli OR therasuit OR “strapping system” OR theratog* OR “suit therapy”) AND “cerebral palsy” AND gait	7
MEDLINE/PubMed	(“suit wear” OR “space suits” OR lycra OR Adeli OR therasuit OR “strapping system” OR theratog* OR “suit therapy”) AND “cerebral palsy” AND gait	12
TOTAL		168

**Table 2:** Summary table of articles included in the systematic review.

Authors Year GR, LoE	Design	Sample	Age (Aver)	Type of CP/GMFCS	Measurement/Tools	Performance of Evaluation	N Sessions, Temporality	Results
Jung et al. 2021 [57] A, 1b	RCT Cross-over	*n* = 24 TFB1 = 24 TFB2 = 24	6.17	Spastic diplegia (I–II)	STV: walking speed, cadence, step length, stride length, single support time, double support time MT: 3D-MAS	Pre/Post-intervention	1 ses (30 m	Post-TFB intervention, showed significant improvements in walking speed, cadence, step length, stride length, single support time, and double support time
Skublewska-Paszkowska et al. 2018 [58] C,4	EDS	*n* = 1 TTO = 1	5	Spastic diplegia (II)	STV: walking speed, cadence, step time, step length MT:3D-MAS	Pre/Post-intervention (w-wh TTO in both cases)	60 ses, 12 week	Significant improvement measured post-intervention without TTO in walking speed. Significant improvement measured post-intervention with TTO in stabilisation of step length
Degelaen et al. 2016 [33] B,3b	QED	*n* = 31 CP = 15 TDC = 16	7.27	Spastic diplegia (I–II)	STV: walking speed, cadence, step length MT:3D-MAS	Own clothes/ wearing FBS	1 ses	CP group wearing FBS, showed significant improvements in walking speed (*p* < 0.003) and cadence (*p* < 0.001), but not in step length
Kim et al. 2016 [59] A, 1b	RCT	*n* = 17 AST/NDT = 8 NDT = 9	5.64	Spastic diplegia, cuadriplegia (I–II)	STV: walking speed, cadence, step length, stride length, step length, and stride length symmetry MT:3D-MAS	Pre/Post-intervention	AST/NDT: 60 ses AST: 30 m/ses,1 ses/day, 5 days/week, 6 weeks NDT: 30 m/ses, 2 ses/day, 5 day/week, 6 weeks NDT: 60 ses 30 m/ses, 2 ses/day, 5 day/week, 6 weeks	AST + NDT group showed significant increase in walking speed, cadence, and step length symmetry (*p* < 0.001, 0.003, 0.027) NDT group improved step length and stride length symmetry (*p* = 0.006, 0.018) None of the groups showed improvement in stride length
Lee 2016 [60] C,4	CS	*n* = 2 AST = 2	4.5	Spastic diplegia (II)	STV: walking speed, cadence, step length, stride length MT: 3D-MAS	Pre/Post-intervention	20 ses (60 m), 5 day/week, 4 weeks	Both cases showed improvement in cadence, only one showed improvement in step length symmetry and walking speed. Results for step length and stride length were variable in each case.
Chang et al. 2015 [61] A, 1b	RCT Cross-over	*n* = 9 ESO 1 = 9 ESO 2 = 9	8.73	Spastic diplegia (I–II)	STV: walking speed, cadence, stride length MT: 3D-MAS	Own clothes/ wearing ESO	1 ses (10 m)	Significant changes between ESO control, showed an increase in walking speed (*p* = 0.036), cadence (*p* = 0.005), and stride length (*p* = 0.011), wearing ESO
Abd El-Kafy 2014 [62] A, 1b	RCT	*n* = 57 NO = 18 TTG = 16 TTG + AFO = 17	7.3	Spastic diplegia (I–II)	STV: walking speed, cadence, stride length MT: 3D-MAS	Pre/Post-intervention	60 ses (2 h), 1 ses/day, 5 days/week, 12 weeks	Significant post-intervention differences among three groups in walking speed, stride length, and cadence (*p* = 0.03, 0.001, 0.011). Best improvements of all gait parameters were shown in group C
Ko et al. 2014 [63] C,4	EDS	*n* = 1 AST = 1	8	Spastic diplegia (III)	STV: walking speed, stride time, stride length, stride width MT: 3D-MAS, 10-MWT	Pre/Post-intervention (1–25 ses) 10-MWT: each ses	18 ses (50 m), 1 ses/day, 1 day/week, 18 weeks	Significant improvement post-treatment in walking speed, stride length, stride width, and step time. 10-MWT improved after each session and growing up until the end of intervention.
Christy et al. 2012 [35] C,4	QED Follow up	*n* = 17 TST = 17	7.6	Spastic, hypotonic, athetoid, ataxic diplegia cuadriplegia triplegia (I–III)	STV: cadence MT: step activity monitor	Pre/Post-intervention (I. effects, 3 months/later)	15 ses (4 h), 1 ses/day, 5 day/week, 3 weeks	Immediate post-intervention and follow-up showed trends of improvements in cadence, but there was no significant improvement immediately after or at 3 months compared to pre-intervention
Bailes et al. 2010 [34] C,4	CS	*n* = 2 TST = 2	7.5	Spastic diplegia (III)	STV: walking speed, cadence, single support time, double support time, step length, stride length MT: 3D-MAS	Pre/Post-intervention (4 d/later)	15 ses (4 h), 1 ses/day, 5 days/week, 3 weeks	Both subjects showed post-intervention improvement in walking speed and cadence. Only one had changes in double support time and single support time. No significant changes were reported in stride length or step length in any case
Matthews et al. 2009 [32] C,4	CS	*n* = 8 DEFO = 8	5.5	Spastic diplegia (I–III)	STV: walking speed MT: 10-MWT	Pre/During/ Post-intervention (Immediate effects and 6 w/later)	30 ses (8 h) 1 ses/day, 5 day/week 6 weeks	Five subjects showed significant changes in walking time and four in walking time variation, between pre-intervention and during intervention in both cases. However, changes were not uniform
Flanagan et al. 2009 [31] C,4	QED	*n* = 5 TTG = 5	7–13	Spastic diplegia (I)	STV: walking speed, cadence, step length, stride length MT: 3D-MAS	Pre/Post-intervention (I. effects with/without suit, 2 and 4 months/later without suit)	84 ses, 1 ses/day, 5 days/week, 12 weeks	There were no significant pre/post-intervention changes in spatio-temporal gait parameters

(AFO) Rigid foot and ankle orthosis; (AST) Adeli suit treatment; (CP) Cerebral palsy; (CS) Case series; (d) Day; (DEFO) Elastomeric tissue dynamic orthosis; (EDS) Experimental design of a subject; (ESO1) External strap orthosis evaluation 1; (ESO2): External strap orthosis evaluation 2; (FBS) Full-body suit; (GR) Grade of Recommendation; (GMFCS) Gross Motor Function Classification System; (h) Hours; (LoE) Level of Evidence; (m) Minutes; (3D-MAS) Three-dimensional movement analysis system; (MT) Measurement tool; (10-MWT) Ten-meter walk test; (N) Total number of patients; (NDT) Neurodevelopmental treatment; (NO) No orthosis; (QED) Quasi-experimental design; (RCT) Randomised clinical trial; (ses) sessions; (STV) Spatio-temporal variables; (TDC) Typically developing children; (TFB 1) Theratogs full body evaluation 1; (TBF 2): Theratog full body evaluation 2; (TST) Therasuit; (TTG) Theratog; (TTO) T-shirt and trouser orthosis; (w) Weeks.

**Table 3:** Evaluation of the methodological quality of the selected studies according to the CMSQ on the effects of the DSO on the spatio-temporal parameters of gait in subjects with cerebral palsy.

	Jung et al. 2021 [57]	Skublewska-Paszkowska et al. 2018 [58]	Degelaen et al. 2016 [33]	Kim et al. 2016 [59]	Lee 2016 [60]	Chang et al. 2015 [61]	Abd El-Kafy 2014 [62]	Ko et al. 2014 [63]	Christy et al. 2012 [35]	Bailes et al. 2010 [34]	Matthews et al. 2009 [32]	Flanagan et al. 2009 [31]
Study quality	7	6	6	8	5	7	7	7	9	7	6	6
External validity (study bias)	0	0	0	1	0	0	1	0	1	0	1	1
Internal validity (study bias)	7	2	4	9	2	9	9	3	4	5	2	2
Statistical power	5	1	1	5	1	5	5	1	5	1	5	3
Total score	19	9	11	23	8	21	22	11	19	13	14	12
Percentage (%)	59	28	34	71	25	65	68	34	59	40	43	37

Percentage of the maximum score (32 points).

## Data Availability

No new data were created in this study. Data sharing is not applicable to this article.
